# Awareness, Preparedness, and Attitudes Toward Lightning Injury Management Among Healthcare Workers in a Tertiary Care Teaching Hospital in Eastern India: A Cross-Sectional Study

**DOI:** 10.7759/cureus.106245

**Published:** 2026-03-31

**Authors:** Mukunda C Sahoo, Biswajeevan Sahoo, Md Sameer

**Affiliations:** 1 Hospital Administration, All India Institute of Medical Sciences, Bhubaneswar, Bhubaneswar, IND

**Keywords:** awareness for prevention, capacity building, lightning injury, management of lightning injury, natural disaster

## Abstract

Background

Incidents of lightning injuries are commonly reported in the coastal state of Odisha, India. It is a major weather-related cause of death in this region. Inadequate awareness of healthcare workers (HCWs) about lightning injury prevention and management can lead to suboptimal patient management. Despite several measures taken by various governments, fatalities due to lightning continue to rise. Injuries from lightning are preventable with adequate awareness and knowledge of the management of such patients. Identifying the knowledge gaps is the first step towards strengthening overall preparedness for lightning injury management in a healthcare organization. This study aims to assess the awareness and preparedness of HCWs in a tertiary care teaching hospital of Eastern India.

Methods

A cross-sectional study was conducted among HCWs in the 960-bed study hospital. Participants (n=246) were selected with a convenient sampling method that included doctors, nurses, technicians, hospital attendants, and other hospital support staff to ensure heterogeneity. Senior faculty members and administrative staff were excluded. A validated questionnaire was developed and provided to participants to assess awareness, attitudes, and preparedness regarding the prevention and management of lightning injuries. A scoring system of one and zero, along with a five-point Likert scale, was used to analyze the responses. The criteria were set to assess awareness level: high awareness (≥70%), moderate (40%-70%), and low awareness (<40%). Data were statistically analyzed using the IBM SPSS Statistics Software, trial version 25 (IBM Corp., Armonk, NY, USA).

Results

The mean awareness score of HCWs was 7.4 out of 12, indicating moderate awareness. Only 39% of participants demonstrated high awareness, with doctors scoring higher than all others. Prior training in emergency healthcare services and the profession of the HCWs were factors associated with high awareness (adjusted OR (aOR) = 3.46, CI = 95%) among the HCWs; 96% of participants felt the need for a structured training program on lightning prevention and the management of affected patients.

Conclusion

The study demonstrated that HCWs have moderate awareness and preparedness for managing lightning injuries. Attitudes toward learning were overwhelmingly positive, with a demand for structured training initiatives. Doctors and those with prior emergency or disaster management training had significantly higher awareness, reaffirming the need for training interventions. However, being conducted in a single setting, the generalizability of the study findings to other settings is limited.

## Introduction

Lightning is a natural phenomenon associated with thunderstorms that is beautiful as well as fatal if it strikes any person or impacts human settlements [1]. From a public health perspective, lightning is a significant environmental hazard, particularly affecting outdoor activities, and is particularly relevant in India, given its population density and agricultural occupation [2]. It transmits a massive atmospheric electrical discharge between clouds and the ground, generating temperatures up to 30,000°C [3], and such intense electrical currents can cause severe injury or death. Tall objects such as trees and buildings are at higher risk of strikes due to their proximity to the discharge pathway and thereby pose a potential threat during a thunderstorm.

Worldwide, lightning occurs approximately 50 times per second, with about 20% of these strikes resulting in direct contact with the ground. Globally, the death toll due to lightning strikes is approximately 24,000 annually [4, 5], and the injuries associated are multifold [6]. The majority of the injuries are preventable with appropriate response mechanisms [7]. 

In India, lightning accounts for nearly 2,500 deaths every year, with states of Odisha, Bihar, Jharkhand, and West Bengal contributing 70% of these fatalities [8, 9]. The state of Odisha, in particular, is among the most lightning-prone [10], with a sharp rise in strikes in recent years. Despite the burden of lightning injuries in the region, it is a rare clinical presentation, as the victims often lose their lives before reaching the healthcare center, leading to limited exposure among healthcare professionals. 

The most common mechanism of lightning injury is through contact or ground currents, rather than direct strikes or side splashes. Although such injuries are often fatal, they are largely preventable through awareness and timely safety measures [11, 12]. The Government of India launched the Lightning Resilient India Campaign in 2019, in collaboration with the Climate Resilient Observing Systems Promotion Council (CROPC), the India Meteorological Department, and the Ministry of Earth Sciences, which has started national-level mitigation programs to reduce the impact of lightning on human life through awareness and preparedness among the general public and healthcare workers (HCWs) [13]. Initiatives such as Damini, a mobile application, provide real-time lightning alerts and safety guidance to the public [14]. Injuries related to lightning are insufficiently available in routine clinical training and emergency response systems. While preventive strategies are increasingly recognized, there is comparatively less emphasis on clinical preparedness within healthcare institutions.

Lightning injuries pose a significant yet under-recognized risk in India. Odisha, a coastal state in India located along the Bay of Bengal, experiences frequent thunderstorms during the pre-monsoon and monsoon seasons due to local meteorological phenomena called Kalbaishakhi storms that are associated with lightning [15]. In general, lightning injury care is supportive and includes cardiac resuscitation for the seriously injured, but patients should ideally be hospitalized and treated as soon as possible [16, 17]. Fewer HCWs have experience managing lightning injuries, as they are rarer than common electrical injuries [18]. By being aware of the basics of lightning and the pathophysiology of injuries associated with lightning strikes, HCWs are better prepared to assess the victim, anticipate complications, and provide effective medical care [19, 20]. 

Despite reports of notable cases of lightning injuries in India, research studies on HCWs' awareness of lightning injuries in India are limited. The objective of this study is to assess the awareness, preparedness, and attitude of HCWs regarding lightning and the management of lightning-related injuries in a tertiary care teaching hospital in Eastern India through a survey in the lines of the Knowledge-Attitude-Practice (KAP) model that explores the very need for targeted training intervention or developing capacity-building strategies for reducing mortality and improving health system resilience to the lightning hazard.

## Materials and methods

The study setting is All India Institute of Medical Sciences, Bhubaneswar, a public-sector tertiary care teaching institution of national importance in Eastern India, which serves as a referral center and a training hub for hospital preparedness in healthcare emergencies, supported by the Disaster Management Cell, Ministry of Health and Family Welfare, Government of India. It is associated with a 960-bed tertiary hospital with all functional specialty and super-specialty departments. The study duration was from October to December 2025. The primary objective of assessing awareness and preparedness for lightning injury management among HCWs across various clinical departments of the institute.

The cross-sectional observational study design was adopted. A non-probability convenience sampling technique was employed to recruit study participants from an overall workforce of approximately 1,000 HCWs. A convenient sampling technique was considered due to the operational nature of the research work. However, multiple cadres of HCWs were included from various departments to maintain the sample's heterogeneity. Similarly, the data collection was performed in lean hours and multiple shifts and days to reduce the selection bias. The eligible and available staff working in various departments were invited to participate in the study. The participants with a minimum of six months of work experience in the hospital were approached department-wise. Senior faculty members and administrative staff were excluded from the study, considering the fact that the senior faculty members are expected to have had greater exposure and training due to their work experience, and the administrative staff are not engaged in patient care activities. Institutional ethical clearance was obtained prior to commencement of the study (approval number: T/IM-NF/Hos.Admin/25-26/189), and written informed consent was secured from all participants.

The sample size was calculated to be 278 using OpenEpi software (Open Source Epidemiologic Statistics for Public Health, Atlanta, GA, USA) with a 95% confidence interval. A total of 300 printed questionnaires were distributed to the staff members, and responses were collected. Two hundred and forty-six responses were collected from participants, constituting the final sample, comprising 246 HCWs, which included resident doctors, nurses, technicians, hospital attendants, and other support staff engaged in hospital services.

The initial phase of the study involved the development of a structured, questionnaire-based data collection tool (Appendix A) based on available literature in consultation with experts in hospital administration, emergency medicine, and community medicine with expertise in disaster management to ensure content validity. The tool was pilot-tested among 25 HCWs to assess clarity, feasibility, and internal consistency. The attitude scale demonstrated acceptable reliability, yielding a Cronbach’s alpha of 0.78, while the preparedness scale showed good reliability with α = 0.81. The questionnaire comprised sections on demographic characteristics, prior exposure or experience with lightning incidents, knowledge regarding causes and risk factors, safety measures, first aid practices, attitudes, preparedness levels, and perceived training needs. Concerning the awareness, nine questions were included with three-point responses, and three questions were included concerning the attitude and preparedness of staff, for which the responses to the questions were based on a five-point Likert scale [21, 22]. A score of one was assigned for correct responses, and zero was assigned for incorrect and 'Do not know' responses in the awareness section. Each correct response was awarded a score of one, while incorrect responses were scored zero. Similarly, there were three questions on practices and training needs and one open-ended question. Awareness levels were categorized as high (≥70%), moderate (40%-70%), and low (<40%) based on cumulative scores.

Data collection was conducted using printed questionnaires. Completed forms were scrutinized for completeness, and incomplete responses were excluded prior to analysis, resulting in a final sample size of 246. Data were entered into Microsoft Excel (Microsoft Corp., Redmond, WA, USA) and subsequently analyzed using the IBM SPSS Statistics Software, trial version 25 (IBM Corp., Armonk, NY, USA). Frequencies and percentages with summarized and composite scores were calculated. Inferential statistics were used to assess the association of independent variables and categorical variables. Logistic regression analysis was performed to identify the factors associated with awareness levels. Descriptive statistics were used to summarize participant characteristics and response distributions. The outcome variables were the awareness level, attitude towards lightning injury prevention and management, preparedness for it, and perceived training needs. Associations between categorical variables were assessed using the chi-square (χ²) test, with a p-value of <0.05 considered statistically significant.

## Results

A total of 246 HCWs participated in the study (Table 1), with doctors at 21.1% (n=52), nurses at 23.6% (n=58), technicians at 26% (n=64), allied health staff at 17.1% (n=42), and other support staff at 12.2% (n=30); 37.4% of participants were below the age of 30 years, with 35.8% in the age group of 30-39 and 26.8% above 40 years. Female participants (54.5%) were higher in number than males (45.5%). The majority of the participants (n = 101) had work experience of less than five years; 84 had five to 10 years of experience, and 61 had worked for more than 10 years in the institution. Only 29.3% (n=72) of respondents had some kind of prior training in disaster management, as compared to 70.7% (174) who did not undergo any training on disaster management, which is more than twice that of participants who did. The mean knowledge score (Table 2) was 7.47 ± 1.8, with 39% having high awareness, 43.9% moderate, and 17.1% having a low level of awareness. Among the participants, 89.4% knew that lightning is a strong atmospheric electric discharge. Also, 91.9% of participants correctly mentioned standing below a tree as an unsafe practice, as compared to 78.5% considering concrete buildings as safe shelters. Among the participants, 86.6% were correct about the consequences of a lightning injury, such as burns, cardiac arrest, death, and other conditions. As for responses, 71.5% correctly mentioned starting cardiopulmonary resuscitation (CPR) for unresponsive victims in a safe place. Among the participants, 83.7% mentioned that using mobile phones increases the risk of exposure to lightning. Only 32.5% of participants knew about the concept of reverse triaging, while 49.6% knew about the 30-30 safety rule. Additionally, 64.7% of participants correctly answered whether a lightning victim can be touched or not.

**Table 1:** Sociodemographic profile of participants (n = 246)

Variable	Category	Frequency (%)
Age (years)	<30	92 (37.4)
	30–39	88 (35.8)
	≥40	66 (26.8)
Gender	Male	112 (45.5)
	Female	134 (54.5)
Profession	Doctor	52 (21.1)
	Nurse	58 (23.6)
	Technician	64 (26.0)
	Allied Health Staff	42 (17.1)
	Support/Other Staff	30 (12.2)
Years of experience	<5 years	101 (41.1)
	5–10 years	84 (34.1)
	>10 years	61 (24.8)
Prior emergency/disaster training	Yes	72 (29.3)
	No	174 (70.7)

**Table 2:** Correct responses to individual awareness items Mean knowledge score: 7.47 ± 1.8 (out of 9); Using pre-defined categories: High awareness (≥70%), 96 (39.0%); Moderate awareness (40%–69%), 108 (43.9%); Low awareness (<40%), 42 (17.1%)

Knowledge Item	Correct Responses (%)
Lightning caused by atmospheric electrical discharge	89.4
Standing under trees during lightning is unsafe	91.9
Mobile phone use outdoors risks exposure to lightning injury	83.7
Concrete buildings are safe shelters	78.5
Victims can be safely touched (not charged)	64.2
30–30 safety rule known	49.6
Lightning can cause burns, cardiac arrest, and neurological injury	86.6
CPR should be initiated immediately	71.5
Reverse triage principle known	32.5

Concerning the attitude and perceived preparedness (Table 3), 95.5% of participants mentioned the need for mandatory training on lightning management for HCWs. Among the participants, 92.3% agreed that lightning injuries can be prevented with awareness. Only 58.9% of participants were confident in providing first aid to lightning victims, and only 46.7% of participants mentioned that the hospital is adequately prepared to handle lightning injuries. The overall mean attitude score was calculated to be 3.98 ± 0.6 from the responses based on the five-point Likert scale. Table 4 shows that the factors of profession (χ² = 14.82, p-value = 0.005), prior emergency or disaster training (n = 72) (χ² = 11.63, p-value = 0.001), and work experience (χ² = 4.02, p-value = 0.045) were associated with comparatively higher awareness about lightning management.

**Table 3:** Attitude and perceived preparedness of the participants

Statement	Agree/Strongly agree (%)
“I feel confident to provide first aid to lightning victims.”	58.9
“Lightning injuries are preventable through awareness.”	92.3
“My hospital is adequately equipped to handle lightning injuries.”	46.7
“Training on lightning management should be mandatory.”	95.5
Overall mean attitude score: 3.98 ± 0.6 (on a 5-point Likert scale) [23]	

**Table 4:** Factors associated with adequate awareness of lightning prevention and injury management among healthcare staff *The value is for the total profession category. **The value is for the prior training category. χ²: chi-square

Factors associated with adequate awareness			
Variable	Adequate Awareness (%)	χ²	p-value
Profession		14.82*	0.005
Doctor (n = 52)	34 (65.4)		
Nurse (n = 58)	29 (50.0)		
Technician (n = 64)	19 (29.7)		
Allied/Support (n = 72)	14 (19.4)		
Prior emergency/disaster training		11.63**	0.001
Yes (n = 72)	49 (68.1)		
No (n = 174)	47 (27.0)		
Experience (>10 yrs)		4.02	0.045
Gender		1.22	0.27

HCWs with prior emergency or disaster management training (adjusted OR (aOR) = 3.46) and the profession of the participant (aOR = 2.87) were the significant predictors of awareness on the management of patients injured by lightning strikes (Table 5).

**Table 5:** Significant associations identified for awareness in lightning management

Factors associated with higher awareness	Adjusted OR (95% CI)		p-value
Doctor vs. other cadres	2.87 (1.31–6.29)		0.008
Prior emergency/disaster training	3.46 (1.81–6.60)		<0.001

## Discussion

The study identified that the awareness level of lightning injury prevention and management among HCWs is moderate, and HCWs, such as resident doctors with prior emergency or disaster management training, showed significant awareness. General awareness about lightning safety practices was reasonably high and was attributed to the social awareness campaign across various media. However, critical knowledge related to clinical management and emergency response was lacking among a large proportion of participants. Considering the exclusion criteria, two-thirds of respondents were under 40 years, reflecting relatively young study participants, with females slightly outnumbering the males. The doctors and nurses are a group of HCWs who are generally more exposed to patient management related to the working duration.

Only one-third of the total participants had received some form of training in disaster or emergency management, highlighting a training gap in lighting management for HCWs. The Government of India has realized the importance of preparing the workforce for lightning management along with public awareness for the prevention of injuries due to lightning [24]. As stated earlier, the occurrence of lightning is very high globally, and eastern India is more prone to fatalities or injuries due to lightning. The National Disaster Management Authority (NDMA), Government of India, started the Lightning Resilient India Campaign to mitigate the morbidity and mortality from lightning injuries through improved awareness and preparedness. It engages the community for better outcomes in terms of the prevention of lightning injuries [14]. Findings of the current study reinforce this by identifying the awareness gap among the HCWs. The majority of the participants of this study had adequate awareness about the possible risks of being struck by lightning and how to prevent it. However, a considerable number of participants were not confident about the management protocol of lightning victims, such as reverse triaging and cardiopulmonary resuscitation, which emphasizes the institutional requirement of training the staff on emergency management of such patients.

A majority of the participants expressed the need for an institutional capacity-building training program on lightning management and prevention, and also for the development of standard operating procedures or guidelines specific to the institutional setting. The lack of such programs possibly correlates with the knowledge levels in lightning injury patient management as observed in this study. Knowledge gaps were identified in the areas of the concept of reverse triaging as well as preventive measures. Reverse triaging is a strategy to optimize healthcare resources during emergency situations like disasters. In such scenarios, the stable patients are being discharged early. It helps to manage the surge capacity by making resources available for more serious patients. It frees up beds and also the manpower engaged by identifying the patients who require minimal care. This also includes the patient who appears dead and non-salvageable. In a similar study by Justice et al. [25], the importance of reverse triaging in optimizing hospital resource allocation during mass casualty incidents was emphasized, which aligns with the observed knowledge gap in this study.

This study reaffirms that training on lightning management should be mandatory for all HCWs, and over 92% of participants believed that awareness could prevent lightning-related injuries. The 30-30 rule [26] for prevention of lightning injury states that if the time between seeing the lightning and hearing thunder is 30 seconds or less, one should immediately seek shelter. The second 30 is the waiting time after the last thunder before resuming outdoor activities. Ironically, about half of the participants were unaware of this rule in view of lightning safety, which is crucial for a timely emergency response. Similar results were observed in a study by Rahman et al. [27] conducted in Malaysia, which highlighted limited awareness of patient management and triage after lightning events.

Another area of concern was noted in participants' confidence in providing first aid to lightning victims, with about 40% not being confident. About half of the participants were of the opinion that the institution is adequately prepared for handling lightning victims. These findings highlight the urgent need to strengthen the infrastructure as well as the human resources for making them ready for handling lightning-struck victims and also protecting themselves or anyone in such an event of potential lightning injury. The study findings were consistent with those conducted by Mishra et al. [28], who mention that only one-third of the public are able to identify the lightning injury prevention practices. In the preventive context, the doctors demonstrated higher awareness than other staff categories, likely due to differences in educational background and better exposure to emergency medical management. Participants in the present study were able to recognize safe and unsafe practices and other common outcomes due to lightning injuries, indicating a good knowledge of lightning hazards. The study correlates the research work of Jensen et al. to understand the importance of preparedness required among HCWs in terms of infrastructure and training in managing lightning victims to improve outcomes [4].

Several studies have been conducted in the past to understand the awareness of the public on lighting injuries; however, this study primarily engages HCWs to assess their awareness level. Malaysia and Bangladesh also report similar weather conditions to those of the study setting in terms of the occurrence of lightning events. Nazri et al. [29], in their study in Malaysia, speak about a moderate level of awareness of lighting safety among the general public. Similarly, Islam and Schmidlin [30], in their study in Bangladesh, concluded that the lack of knowledge and preventive awareness in the case of lightning is a major cause of fatality or death due to lightning. Concerning the HCWs with prior disaster management training exhibiting significantly higher awareness, reinforcing the importance of structured training interventions, the study institution already conducts periodic training on hospital preparedness for healthcare emergencies, during which participants are trained, evaluated, and subsequently tasked with replicating these programs in their respective departments as part of institutional capacity-building. This framework can be further strengthened by incorporating lightning injury management modules, given the growing frequency of lightning events in Odisha.

In another study, Mills et al. [31] highlight the high fatality rates due to lightning, reiterating the fact that it is a major natural hazard in Odisha, and emphasize the necessity of structured training programs for healthcare staff, and their findings directly correlated with the present study. Similarly, in another study by Mishra et al. [32], the lightning-prone zones across Odisha were mapped, identifying vulnerable districts where this tertiary care hospital serves as the nearest or the only referral center. Regular mock drills, inclusion of lightning management modules in emergency training curricula, and public education campaigns are imperative to enhance preparedness. Given the study setting's location in Eastern India, it is ideally positioned to serve as a training and referral center for lightning injury preparedness and management in the region. Currently, it functions as a national training hub for disaster and healthcare emergency preparedness under the guidance of the Disaster Management Cell, Ministry of Health and Family Welfare, Government of India.

Although Odisha has implemented a comprehensive State Disaster Management Program, lightning has not received the same attention as cyclones or floods [33,34]. One cannot reduce the occurrences of lightning incidents, being a natural phenomenon, but preparedness and awareness can lead to a reduction in mortality due to lightning strikes. Therefore, there is a need to ensure infrastructure preparedness for lightning injury management in the region. Strengthening HCWs' awareness through hospital training programs and community-based awareness programs in collaboration with the NDMA can significantly improve readiness and also reduce the burden of lightning-related injuries. The findings of this study strongly support integrating lightning injury management training into disaster response programs for all categories of HCWs [35]. Replication of this study in smaller healthcare centers in the lightning-prone regions is also recommended.

Strengths and limitations

Inclusion of multiple cadres of HCWs to assess awareness of the prevention and management of lightning-related injuries is considered a strength. The methodology adopted provided a comprehensive assessment of institutional awareness, but also initiated the need for structured training across all staff levels. This study adds to the novel effort of the Government of India to evaluate and enhance preparedness for lightning-related emergencies, which is an area often overlooked in healthcare training. The study was conducted at a single healthcare center, limiting the generalizability of the findings to other settings. The convenience sampling technique is also considered a limitation in this study. The self-reported nature of responses may introduce reporting bias, and the study did not explore underlying barriers contributing to low-to-moderate awareness levels. As a way forward, it is planned to include interventional training studies and qualitative assessments to better understand these barriers and assess the impact of targeted educational programs.

## Conclusions

The study demonstrated that HCWs have moderate awareness and preparedness for managing lightning injuries. Attitudes toward learning were overwhelmingly positive, with a demand for structured training initiatives. Doctors and those with prior emergency or disaster management training had significantly higher awareness, reaffirming the value of formal training interventions. The findings highlight a need for a comprehensive training program for healthcare workers and the inclusion of lightning management in disaster preparedness content that can collectively improve readiness and patient outcomes in lightning-related emergencies.

## Appendices

Appendix A 

**Table 6:** Study questionnaire

Questionnaire:
Demographics
1. Age (years): ___
2. Gender: ☐ Male ☐ Female ☐ Other
3. Designation: ☐ Doctor ☐ Nurse ☐ Technician ☐ Allied staff
4. Years of experience: ☐ <5 ☐ 5-10 ☐ >10
5. Department:
6. Previous training in disaster / lightning injury management? ☐ Yes ☐ No
Awareness on Lightning
7. Lightning is caused by accumulation and discharge of electrical energy in the atmosphere. ☐ True ☐ False ☐ Don’t know
8. Standing under trees during lightning is safe. ☐ True ☐ False ☐ Don’t know
9. Using mobile phones outdoors during lightning increases risk. ☐ True ☐ False ☐ Don’t know
10. Staying inside a reinforced concrete building is safe during lightning. ☐ True ☐ False ☐ Don’t know
11. Lightning victims are electrically charged and unsafe to touch. ☐ True ☐ False ☐ Don’t know
12. The “30-30 rule” helps in deciding when to seek shelter. ☐ True ☐ False ☐ Don’t know
13. Lightning can cause burns, cardiac arrest, and neurological injury. ☐ True ☐ False ☐ Don’t know
14. CPR should be initiated immediately for an unconscious lightning victim. ☐ True ☐ False ☐ Don’t know
15. Reverse triage means apparently dead lightning victims should receive first resuscitation. ☐ True ☐ False ☐ Don’t know
Attitude and Preparedness
16. I feel confident to provide first aid to a lightning injury victim. ☐ Strongly agree ☐ Agree ☐ Neutral ☐ Disagree ☐ Strongly disagree
17. My hospital is adequately equipped to manage lightning injuries. ☐ Strongly agree ☐ Agree ☐ Neutral ☐ Disagree ☐ Strongly disagree
18. Lightning injuries are preventable through community awareness. ☐ Strongly agree ☐ Agree ☐ Neutral ☐ Disagree ☐ Strongly disagree
Training Needs
19. Have you ever attended a session on lightning or electrical injury management? ☐ Yes ☐ No
20. Would you like future training on lightning injury management? ☐ Yes ☐ No
21. Have you ever managed or witnessed a lightning victim? ☐ Yes ☐ No
Open-Ended
22. What measures do you suggest to improve awareness on lightning safety and management in hospitals?
